# Predictors of Treatment Outcomes Among HIV-Positive Patients with Drug-Resistant Tuberculosis in Rural Eastern Cape, South Africa: A Retrospective Cohort Study

**DOI:** 10.3390/ijerph23040474

**Published:** 2026-04-09

**Authors:** Thembile Zini, Urgent Tsuro, Lindiwe Modest Faye, Ncomeka Sineke, Monwabisi Faleni

**Affiliations:** 1 School of Public Health, Faculty of Medicine and Health Sciences, Walter Sisulu University, Private Bag X1, Mthatha 5100, South Africa; tzini@wsu.ac.za; 2 WSU TB Research Group, School of Pathology, Faculty of Medicine and Health Sciences, Walter Sisulu University, Private Bag X1, Mthatha 5100, South Africa; utsuro@wsu.ac.za (U.T.); 209101237@mywsu.ac.za (N.S.)

**Keywords:** drug-resistant tuberculosis, XDR-TB, MDR-TB, HIV co-infection, treatment outcomes, predictors, rural health, Eastern Cape, logistic regression, public health

## Abstract

**Highlights:**

**Public health relevance—How does this work relate to a public health issue?**

- The study focuses on the increased prevalence of drug-resistant tuberculosis (DR-TB) among HIV-positive people in rural South Africa, which is a major public health concern linked with high mortality, delayed treatment, and continuous transmission.
- By identifying clinical and socioeconomic predictors of treatment outcomes among MDR-TB and XDR-TB patients, the study adds evidence to improve TB-HIV programme management in high-burden rural areas.

**Public health significance—Why is this work of significance to public health?**

- The study observed that XDR-TB is the biggest predictor of poor treatment outcomes, emphasising the serious impact of advanced drug resistance on TB control and patient survival.
- The study additionally discovered that socioeconomic factors, including financial level, had an impact on treatment results, emphasising the importance of both clinical care and social support interventions in tuberculosis management.

**Public health implications—What are the key implications or messages for practitioners, policy makers and/or researchers in public health?**

- Early molecular resistance testing and enhanced management of XDR-TB patients should be prioritised in TB programmes, especially in HIV-co-infected populations in rural areas.
- To improve treatment success and lessen the burden of DR-TB, policymakers and researchers should strengthen integrated TB-HIV services as well as social protection programmes (such as adherence support and socioeconomic aid).

**Abstract:**

Background: Drug-resistant tuberculosis (DR-TB) remains a major public health challenge in South Africa, particularly in rural settings with high HIV co-infection rates. Understanding predictors of treatment response among people living with HIV is essential for improving clinical management and programmatic outcomes. This study aimed to identify socio-demographic and clinical predictors of treatment outcomes among HIV-positive individuals diagnosed with multidrug-resistant (MDR) and extensively drug-resistant tuberculosis (XDR-TB) in rural Eastern Cape Province, South Africa. Methods: A retrospective cohort study was conducted using routinely collected clinical records of DR-TB patients initiated on treatment between January 2020 and December 2024 at two public healthcare facilities. A total of 239 patients with complete treatment outcome data were included. Treatment outcomes were classified as favourable (cured or treatment completed) or unfavourable (death, treatment failure, or loss to follow-up). Descriptive statistics were used to summarise patient characteristics, while univariate and multivariable logistic regression analyses were performed to identify factors associated with treatment outcomes. Results: Most participants were aged ≤ 39 years (58%), male (60%), unemployed (90%), and without income (80%). MDR-TB accounted for 40% of cases, rifampicin-resistant-TB (RR-TB) for 53%, and XDR-TB for 7.1%. Multivariable analysis showed that XDR-TB was the strongest independent predictor of unfavourable treatment outcome (AOR = 0.18; 95% CI: 0.06–0.58; *p* = 0.004). Income status was also significantly associated with outcome, with participants reporting some incomes having lower odds of favourable outcomes (AOR = 0.46; 95% CI: 0.23–0.92; *p* = 0.036). The model demonstrated modest predictive performance (AUC = 0.67). Conclusions: These findings highlight the dominant influence of resistance phenotype, particularly XDR-TB, on treatment prognosis among HIV-positive DR-TB patients in rural Eastern Cape. Integrating early resistance profiling, intensified clinical management of XDR-TB, and socioeconomic support mechanisms may improve treatment outcomes in high-burden rural settings.

## 1. Introduction

Drug-resistant tuberculosis (DR-TB), including rifampicin-resistant (RR-TB), multidrug-resistant (MDR-TB), and extensively drug-resistant tuberculosis (XDR-TB), remains a major threat to global tuberculosis control [1,2]. South Africa is among the countries with the highest burden of both tuberculosis and HIV co-infection, with rural provinces such as the Eastern Cape experiencing disproportionate morbidity and mortality [3,4]. Among people living with HIV (PLHIV), treatment outcomes for DR-TB are frequently compromised by immune suppression, treatment toxicity, drug–drug interactions, and delayed diagnosis [5,6].

A growing body of evidence has identified several predictors influencing treatment outcomes in TB–HIV co-infected populations [3,7]. Clinical factors such as advanced immunosuppression, delayed initiation of antiretroviral therapy (ART), high bacillary burden, and extensive drug resistance are consistently associated with poorer outcomes [8,9]. In particular, XDR-TB significantly reduces treatment success due to resistance to key second-line agents, prolonged treatment duration, and limited therapeutic options [1,10]. Immunological status, often reflected by CD4 cell counts and viral load suppression, further influences treatment response, as severe immune compromise may impair the host’s ability to control Mycobacterium tuberculosis infection during therapy [11].

In addition to biological determinants, socio-demographic and structural factors also shape treatment outcomes. Variables such as age, gender, income status, education level, and employment can influence treatment adherence and access to healthcare [12,13]. Structural challenges including poverty, food insecurity, unstable employment, and limited health literacy may disrupt treatment continuity and contribute to delayed healthcare seeking [14,15]. Clinical history, including previous TB treatment, relapse status, and the extent of drug resistance, further complicates treatment trajectories and has been associated with unfavourable outcomes [16,17].

Despite growing global evidence, context-specific data from rural high-burden settings remain limited. Rural health systems often face additional constraints, including limited diagnostic capacity, delayed access to specialised DR-TB care, and broader socioeconomic vulnerability [18,19]. Identifying predictors of treatment outcomes among HIV-positive individuals with DR-TB in such settings is therefore essential for improving risk stratification, guiding targeted clinical management, and strengthening TB–HIV programme responses.

A growing body of systematic reviews and meta-analyses has shown that HIV co-infection, advanced drug resistance, previous TB treatment, and socioeconomic vulnerability are major drivers of poor treatment outcomes among people with drug-resistant tuberculosis. However, much of this evidence comes from pooled multi-country studies or urban settings, leaving important gaps in our understanding of how these factors operate in rural, resource-limited contexts. In regions with a high HIV burden, such as the Eastern Cape, identifying locally relevant predictors of treatment outcomes is essential for designing effective, context-appropriate programmatic interventions. This study addresses this gap by providing setting-specific evidence on the predictors of treatment outcomes among HIV-positive individuals with drug-resistant TB in rural South Africa. This study aimed to identify socio-demographic and clinical predictors of treatment outcomes among HIV-positive individuals diagnosed with MDR- and XDR-pulmonary tuberculosis in rural Eastern Cape, South Africa.

## 2. Materials and Methods

### 2.1. Study Design and Setting

A retrospective cohort study was conducted using routinely collected clinical records of patients diagnosed with drug-resistant tuberculosis (DR-TB) who were initiated on treatment at two purposively selected public health clinics in Eastern Cape Province, South Africa, between January 2020 and December 2024. The study sites were selected to represent both rural and peri-urban healthcare settings within the province.

During the study period, 385 eligible patients with drug-resistant tuberculosis were started on treatment. All were confirmed to be HIV-positive at the time of DR-TB treatment initiation, based on routine clinical records. Among these patients, 239 had complete socio-demographic, clinical, and treatment outcome information and were included in the final analysis. Records with missing or incomplete outcome data were excluded to preserve the robustness of the analysis. As the study relied on routinely collected programmatic data from selected facilities, the dataset represents a complete census of available records rather than a sampled population; therefore, a conventional participation rate does not apply.

Patients with missing treatment outcome information or incomplete records were excluded. Eligible participants comprised individuals with microbiologically confirmed rifampicin-resistant tuberculosis (RR-TB), multidrug-resistant tuberculosis (MDR-TB), pre-extensively drug-resistant tuberculosis (Pre-XDR-TB), or extensively drug-resistant tuberculosis (XDR-TB). These categories were included to reflect the full clinical spectrum of DR-TB as currently defined by the World Health Organization, thereby enhancing the comparability of findings with global evidence.

The use of retrospective routine programme data enabled the inclusion of a larger sample size and allowed for the assessment of treatment outcomes under real-world clinical and programmatic conditions.

### 2.2. Study Variables

The primary outcome was treatment outcome, classified as favourable (cured or treatment completed) or unfavourable (death, treatment failure, or loss to follow-up). Independent variables included socio-demographic factors (age, sex, education level, income, and occupation), clinical characteristics (comorbidities, previous TB treatment history, patient category, type of resistance, and type of DR-TB), and social history variables.

### 2.3. Statistical Analysis

Descriptive statistics were used to summarise patient characteristics. Categorical variables were presented as frequencies and percentages, while continuous variables were summarised using means and standard deviations or medians and interquartile ranges, depending on the distribution.

Univariate logistic regression was performed to assess the association between each independent variable and treatment group membership. Variables with a *p*-value < 0.20 in univariate analysis were considered for inclusion in the multivariate logistic regression model.

Multivariate logistic regression was then conducted to identify independent factors associated with treatment group membership. Adjusted odds ratios (AORs) with 95% confidence intervals (CIs) were reported. Multicollinearity was assessed using the variance inflation factor (VIF). Model fit was evaluated using Hosmer–Lemeshow goodness-of-fit test and residual deviance. The discriminative ability of the model was assessed using the area under the receiver operating characteristic curve (AUC).

All analyses were performed in R version 4.5.2 (31 October 2025 ucrt), and statistical significance was set at *p* < 0.05.

## 3. Results

### 3.1. Participant Characteristics

**Figure 1:**
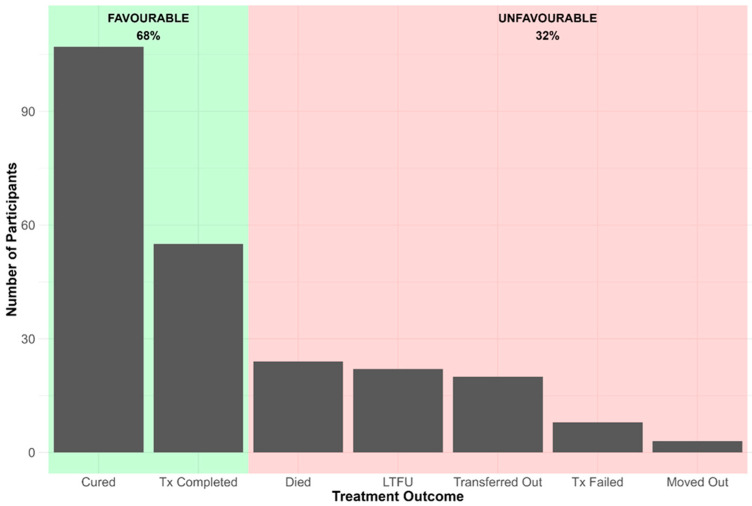
Plot of number of participants by treatment outcome.

A total of 239 participants with complete clinical and treatment outcome data were included in the analysis. In Figure 1 treatment outcomes were classified as favourable (cured or treatment completed) or unfavourable (death, treatment failure, or loss to follow-up).

#### 3.1.1. Socio-Demographic Characteristics

Table 1 presents the distribution of socio-demographic characteristics stratified by treatment outcome (favourable vs. unfavourable). Most participants were aged ≤39 years (58%), male (60%), unemployed (90%), and had no income (80%). The majority had poly-drug resistance (61%) and RR-TB (53%), while 7.1% were diagnosed with XDR-TB.

#### 3.1.2. Age Distribution

The majority of participants were aged ≤39 years (58%). Among those with favourable outcomes, 61% were ≤39 years, compared to 52% in the unfavourable group. Conversely, participants aged ≥50 years constituted a larger proportion of the unfavourable group (22%) compared to the favourable group (15%). This pattern suggests a potential trend toward poorer treatment outcomes among older individuals, although formal statistical testing is required to confirm significance. Participants aged 40–49 years were similarly distributed across favourable (24%) and unfavourable (26%) outcomes, indicating no marked difference in this age category.

#### 3.1.3. Gender

Overall, 60% of participants were male and 40% were female. Males represented a higher proportion of the favourable outcome group (63%) compared to the unfavourable group (53%). By contrast, females constituted a greater proportion of the unfavourable group (47%) compared to the favourable group (37%). This distribution suggests a possible gender-related difference in treatment response; however, the magnitude of difference appears modest.

#### 3.1.4. Education Level

Educational attainment was similar across outcome categories. The majority of participants had medium-level education (70%), followed by low (19%) and high (10%) education. The proportions were nearly identical between favourable and unfavourable groups, indicating no observable association between education level and treatment outcome at the descriptive level.

#### 3.1.5. Income Status

Most participants reported having no income (80%). However, individuals with no income were more frequently represented in the favourable outcome group (84%) compared to the unfavourable group (73%). Conversely, those with some income comprised a larger proportion of the unfavourable group (27%) compared to the favourable group (16%). This pattern suggests a counterintuitive trend whereby participants with some income appeared more likely to experience unfavourable outcomes. This finding warrants further analytical exploration to understand underlying socioeconomic dynamics.

#### 3.1.6. Occupation

The majority of participants were unemployed (90%). Unemployment was slightly more common in the unfavourable group (92%) compared to the favourable group (88%). Employment was more frequent among those with favourable outcomes (12%) compared to those with unfavourable outcomes (7.8%). However, the overall number of employed individuals was small, limiting strong descriptive inference.

#### 3.1.7. Social History

Most participants reported no substance use history (61%). A slightly higher proportion of individuals with no substance use were observed in the unfavourable group (65%) compared to the favourable group (59%). Single substance use was somewhat more common among those with favourable outcomes (28%) than in those with unfavourable outcomes (23%), while multiple substance use was similarly distributed across groups (14% vs. 12%).

#### 3.1.8. Clinical Characteristics

Table 2 summarises the clinical characteristics of participants stratified by treatment outcome (favourable vs. unfavourable).

#### 3.1.9. Previous Drug History

The majority of participants (59%) had a history of previous TB treatment, while 41% were newly treated cases. The distribution was similar between outcome groups: 59% of those with favourable outcomes and 61% of those with unfavourable outcomes had prior treatment history. This suggests that previous TB treatment exposure was common in this cohort but did not demonstrate a marked descriptive difference between outcome groups.

#### 3.1.10. Patient Category

When categorised by patient classification at presentation, new cases constituted 42% of the overall cohort, with a slightly higher proportion observed among those with favourable outcomes (44%) compared to those with unfavourable outcomes (39%). Relapse cases accounted for 40% overall and were marginally more represented in the unfavourable group (42%) than in the favourable group (39%). Treatment failure cases comprised 18% of the cohort, with comparable proportions between favourable (17%) and unfavourable (19%) outcomes. Overall, the distribution of patient categories was relatively balanced across outcome groups, suggesting no strong descriptive association between patient classification and treatment outcome. However, relapse and treatment failure cases were modestly more frequent among individuals with unfavourable outcomes, indicating a possible trend that warrants further analytical assessment.

#### 3.1.11. Type of Resistance (Mono vs. Poly Drug Resistance)

Poly-drug resistance was more common (61%) than mono-resistance (39%) in the overall cohort. Among participants with unfavourable outcomes, 64% had poly-resistance compared to 60% in the favourable group. Conversely, mono-resistance was slightly more common in the favourable group (40%) compared to the unfavourable group (36%). Although the difference is modest, this trend suggests that broader resistance profiles may be associated with poorer outcomes.

#### 3.1.12. Type of DR-TB

The distribution of DR-TB type demonstrated clear differences across treatment outcome groups. Overall, MDR-TB accounted for 40% of cases, with a slightly higher proportion observed among participants with favourable outcomes (42%) than among those with unfavourable outcomes (35%). Similarly, RR-TB constituted 53% of the cohort and was more common in the favourable group (55%) than in the unfavourable group (49%). By contrast, XDR-TB represented only 7.1% of the total cohort but showed a pronounced imbalance between outcome categories, comprising 3.1% of favourable cases and 16% of unfavourable cases. This substantial overrepresentation of XDR-TB among participants with unfavourable outcomes suggests a strong descriptive association between XDR-TB and poorer treatment response.

#### 3.1.13. Comorbidity

Most participants (96%) had a single comorbidity, while only 4.2% had multiple comorbidities. Multiple comorbidities were slightly more frequent in the favourable group (4.9%) than in the unfavourable group (2.6%).

### 3.2. Univariate Analysis

Univariate logistic regression identified several variables meeting the threshold (*p* < 0.20) for multivariable modelling (Table 3). Patients with XDR-TB had significantly lower odds of favourable treatment outcomes compared to those with MDR-TB (OR = 0.17; 95% CI: 0.06–0.50; *p* = 0.002). Participants reporting some income also had reduced odds of favourable outcomes compared to those with no income (OR = 0.51; 95% CI: 0.27–0.95; *p* = 0.043). Age ≥ 50 years (OR = 0.57; *p* = 0.127) and male gender (OR = 1.49; *p* = 0.153) demonstrated non-significant trends toward association with treatment outcome.

### 3.3. Multivariable Logistic Regression

Variables with *p* < 0.20 in univariate analysis (age group, gender, type of DR-TB, income, and comorbidity type) were included in the multivariable model (Table 4).

#### 3.3.1. Type of DR-TB

XDR-TB remained independently associated with significantly reduced odds of favourable treatment outcome compared to MDR-TB (AOR = 0.18; 95% CI: 0.06–0.58; *p* = 0.004). RR-TB did not significantly differ from MDR-TB (*p* = 0.749).

#### 3.3.2. Income

Participants reporting some income had significantly lower odds of favourable outcomes compared to those without income (AOR = 0.46; 95% CI: 0.23–0.92; *p* = 0.036).

#### 3.3.3. Age and Gender

Age ≥50 years showed a borderline association with reduced favourable outcomes (AOR = 0.51; 95% CI: 0.24–1.09; *p* = 0.089). Male gender was not significantly associated with outcome (AOR = 1.55; *p* = 0.146).

#### 3.3.4. Comorbidity Type

Comorbidity type was not independently associated with treatment outcome (*p* = 0.143).

### 3.4. Model Performance

No evidence of multicollinearity was observed (GVIF values ≈ 1 for all predictors) Table 5.

**Figure 2:**
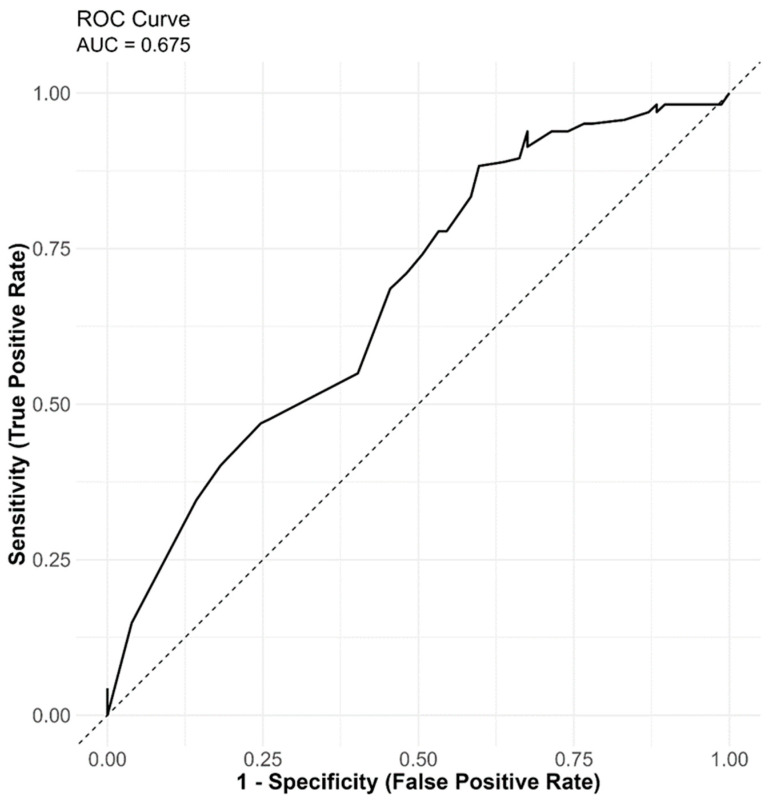
ROC curve for multivariable model predicting DR-TB treatment outcomes. The dotted line indicates that along it the true positives rate equals to the false positive rate at every point, it corresponds to an AUC of 0.5.

Figure 2 illustrates the receiver operating characteristic (ROC) curve for the multivariable logistic regression model predicting treatment outcomes among HIV-positive individuals with drug-resistant tuberculosis in rural Eastern Cape, South Africa. The model showed modest discriminative ability (AUC = 0.67) in distinguishing favourable from unfavourable outcomes and demonstrated adequate calibration based on the Hosmer–Lemeshow goodness-of-fit test (χ^2^ = 12.74, df = 8, *p* = 0.121). No multicollinearity was detected among predictors (VIF < 5).

## 4. Discussion

In this retrospective cohort study of HIV-positive individuals with DR-TB in rural Eastern Cape, we examined socio-demographic and clinical predictors of treatment outcome using both descriptive and multivariable analyses. The discussion is structured to reflect the progression of findings presented in the Section 3.

### 4.1. Descriptive Patterns in Socio-Demographic Characteristics

At the descriptive level, younger participants (≤39 years) were more frequently represented among those with favourable outcomes, whereas older individuals (≥50 years) were proportionally more common in the unfavourable group. Although differences were modest, this pattern suggests a potential age-related vulnerability that may reflect immune senescence, comorbidity burden, or reduced physiological resilience among older HIV-positive patients. These findings are consistent with several studies in Africa and globally suggesting physiological decline associated with aging [20,21,22,23,24]. On the contrary, a study by Hosu et al. suggested that older age improves outcomes, whereas others linked earlier or middle age to better outcomes owing to differences in study populations [25].

Gender differences were observed descriptively, with males more represented in the favourable outcome group and females proportionally higher in the unfavourable group. However, the magnitude of difference was small, suggesting that sex alone may not be a dominant determinant of treatment response in this cohort. By contrast, several studies associated male gender as a predictor of poorer treatment outcomes, which then suggests that differences may be due to variability across regions and cohorts [22,26,27]. This discrepancy in findings encourages the consideration of exploring other predictors, including socioeconomic status, ART adherence, health seeking behaviour and substance use, rather than treating gender solely.

The association observed that the difference between income status and treatment outcomes appeared counterintuitive, with participants reporting some income showing lower odds of favourable outcomes. This finding, however, should be interpreted cautiously. Income was measured using a simple binary classification (“some income” vs. “no income”), which does not capture important dimensions such as income stability, source, or adequacy. In rural settings, having “some income” often reflects informal or unstable employment, which may interfere with treatment adherence because of increased mobility, competing livelihood demands, and irregular access to healthcare services. By contrast, individuals without income may be more likely to access structured social support mechanisms, such as disability grants, which can help support adherence to prolonged treatment. This observation underscores the limitations of relying on simplified socioeconomic indicators to represent complex structural and economic realities.

Educational attainment and occupational status demonstrated minimal variation across outcome categories, suggesting limited descriptive association with treatment outcome. However, a previous cohort study indicated that lower educational level has been associated with increased treatment default, poor treatment adherence, and delayed healthcare seeking behaviours resulting to poorer treatment success [28,29]. This then emphasises the need for structural and patient-centred health literacy interventions.

### 4.2. Descriptive Patterns in Clinical Characteristics

Previous TB treatment exposure was common in the cohort but showed no meaningful imbalance between the favourable and unfavourable groups. Similarly, patient classification (new, relapse, treatment failure) demonstrated relatively balanced distribution across outcomes, although relapse and treatment failure cases were slightly more represented among unfavourable outcomes. Comparably, a recent DR-TB database analysis in Cameroon reported that a history of previous DR-TB was significantly associated with poor treatment outcomes, including increased risk of death or treatment refusal. This suggests that retreatment cases (i.e., treatment exposure) are more vulnerable to unsuccessful outcomes [30].

Poly-drug resistance was marginally more common in the unfavourable group compared to mono-resistance; however, the difference was modest. By contrast, the distribution of DR-TB subtype revealed a pronounced imbalance. XDR-TB represented a small proportion of the overall cohort but was substantially overrepresented among participants with unfavourable outcomes. These findings were like those reported by Safaev et al. and the 2020 Global TB report by the WHO [31,32]. This descriptive signal strongly suggested a clinically meaningful association between XDR-TB and poorer treatment response.

### 4.3. Univariate Associations

Univariate analysis confirmed the descriptive trends. XDR-TB was significantly associated with reduced odds of favourable outcome compared to MDR-TB. Income status was also significantly associated with outcome, with participants reporting some income demonstrating lower odds of favourable response. Age ≥50 years and male gender showed trends toward association but did not reach statistical significance. Other variables, including education level, social history, patient category, and resistance type (mono vs. poly), were not significantly associated with outcome in univariate models. These findings refined the descriptive observations and identified variables warranting inclusion in multivariable modelling.

### 4.4. Independent Predictors of Treatment Outcome

In multivariable analysis, XDR-TB remained the strongest independent predictor of unfavourable outcome. Patients with XDR-TB had markedly reduced odds of achieving favourable treatment outcomes compared to those with MDR-TB. Correspondingly, a review article emphasised the need for health initiatives aimed at improving outcomes for XDR-TB patients by prioritising XDR-TB prevention activities [33]. This finding is consistent with global evidence demonstrating poorer survival and increased treatment failure among XDR-TB patients due to limited therapeutic options, delayed culture conversion, and higher regimen toxicity [34,35,36].

Income status also remained independently associated with treatment outcome. Participants with some income had lower odds of favourable outcomes compared to those with no income. In rural Eastern Cape, income may reflect informal employment, seasonal work, or labor migration, potentially disrupting treatment continuity. Conversely, individuals without income may qualify for disability grants or structured social support, which may facilitate adherence. These findings were consistent with studies in Uganda, Kenya, Ethiopia, and South Africa suggesting the need for targeted adherence support, risk stratification, and social protection measures [3,24,26,37].

Age ≥50 years demonstrated borderline association in the multivariable model, maintaining the same directional trend observed descriptively. While not statistically significant, the magnitude of effect suggests potential clinical relevance. Moreover, given that age is a non-modifiable disease risk factor, sensitising vulnerable age groups to prevent TB-HIV co-infection may help attenuate its negative consequences [38]. Gender and comorbidity type were not independently associated with outcome after adjustment, indicating that their descriptive differences were likely confounded by other variables.

### 4.5. Model Performance and Implications

The multivariable model demonstrated adequate calibration, as indicated by the non-significant Hosmer–Lemeshow goodness-of-fit test, suggesting that the predicted probabilities were reasonably consistent with the observed outcomes. Additionally, no evidence of problematic multicollinearity was detected among the predictors, indicating that the independent variables included in the model were sufficiently distinct and did not distort the regression estimates. These findings support the internal validity of the model and suggest that the identified predictors were appropriately specified within the analytical framework.

Despite adequate calibration, the model’s discriminative ability was modest, with an area under the receiver operating characteristic curve (AUC) of 0.67. An AUC in this range indicates that the model performs better than chance in distinguishing between favourable and unfavourable treatment outcomes, but its predictive accuracy remains limited. This level of performance is not uncommon in studies using routine programmatic data, where clinical records may lack detailed biological, behavioural, and adherence-related variables that could enhance predictive precision [39]. The modest AUC therefore reflects the complexity of DR-TB treatment outcomes, which are influenced by a multifactorial interplay of biological, clinical, and socioeconomic determinants.

The findings suggest that while key variables such as DR-TB subtype particularly XDR-TB and income status contribute meaningfully to predicting treatment outcomes, additional predictors are likely necessary to strengthen model performance. Variables that were not available in this dataset, such as CD4 cell count, viral load suppression, antiretroviral therapy adherence, treatment regimen composition, time to culture conversion, nutritional status, and adherence support mechanisms, may substantially improve predictive accuracy. These factors are known to influence treatment response among HIV-positive individuals with drug-resistant tuberculosis and may capture aspects of disease severity and treatment engagement not reflected in routine demographic or clinical variables.

From a programmatic perspective, the model nonetheless provides valuable insights into clinical risk stratification. Identifying XDR-TB as a dominant predictor of unfavourable outcome reinforces the need for early molecular resistance profiling and intensified clinical monitoring of patients with highly resistant diseases. Similarly, the observed association with income status highlights the importance of addressing structural determinants of health through social protection, adherence support, and patient centered care interventions. Future predictive modelling efforts in high-burden rural settings should aim to integrate clinical, immunological, behavioural, and health system variables, potentially using larger datasets and advanced modelling approaches such as machine learning to improve predictive performance and support precision public health strategies for DR-TB management.

The modest discriminative ability of the model (AUC = 0.67) suggests that additional predictors not captured in the dataset may play a significant role in determining treatment outcomes. HIV-related clinical markers (e.g., CD4 count and viral load suppression), adherence-related factors, and health system variables are likely important contributors. Their absence underscores the need for more comprehensive data collection in routine programmatic settings.

## 5. Strengths and Limitations

This study has several limitations that should be considered when interpreting the findings. First, reliance on routinely collected retrospective data restricted the availability and level of detail of several key variables. Socioeconomic factors were measured using simplified indicators that may not fully capture income stability, household conditions, or broader structural vulnerability.

Second, several important behavioural and clinical variables, including TB and ART adherence, CD4 cell count, viral load, duration on ART, and the availability of adherence support mechanisms, were not available in the dataset. The absence of these factors likely contributed to the modest predictive performance of the model and limited the ability to identify potentially modifiable determinants of treatment outcomes.

In addition, the retrospective design introduces the possibility of residual confounding, as unmeasured or incompletely captured variables may have influenced the observed associations. This may partly explain why some factors, such as age and gender, showed descriptive differences but did not remain statistically significant after adjustment.

Finally, the study was conducted in two purposively selected facilities within a rural province, which may limit the generalisability of the findings to other settings. Nevertheless, despite these limitations, the study provides valuable real-world insights into DR-TB treatment outcomes among HIV-positive individuals in a high-burden rural context.

## 6. Conclusions

In this cohort of HIV-positive individuals with drug-resistant tuberculosis in rural Eastern Cape, resistance phenotype particularly XDR-TB emerged as the strongest independent predictor of unfavourable treatment outcome, underscoring its dominant clinical influence on prognosis. Socioeconomic context, notably income status, also independently affected outcomes, reflecting the interaction between biological resistance mechanisms and structural determinants of health. These findings emphasise the importance of early molecular resistance profiling, intensified and targeted management of XDR-TB cases, and integration of socioeconomic support within DR-TB treatment programmes. Future predictive models should incorporate immunological markers and adherence-related variables to strengthen risk stratification and support precision public health approaches in high-burden rural settings.

## Figures and Tables

**Table 1:** Socio-demographic characteristics of study participants.

Characteristic	Category	Overall N = 239 ^1^	Favourable N = 162 ^1^	Unfavourable N = 77 ^1^
Age group	≤39	139 (58%)	99 (61%)	40 (52%)
	40–49	59 (25%)	39 (24%)	20 (26%)
	≥50	41 (17%)	24 (15%)	17 (22%)
Gender	Female	96 (40%)	60 (37%)	36 (47%)
	Male	143 (60%)	102 (63%)	41 (53%)
Education	Low	46 (19.2%)	31 (19.1%)	15 (19.5%)
	Medium	168 (70.3%)	114 (70.4%)	54 (70.1%)
	High	25 (10.5%)	17 (10.5%)	8 (10.4%)
Income	No income	192 (80%)	136 (84%)	56 (73%)
	Some income	47 (20%)	26 (16%)	21 (27%)
Occupation	Employed	25 (10%)	19 (12%)	6 (7.8%)
	Unemployed	214 (90%)	143 (88%)	71 (92.2%)
Social history	None	145 (61%)	95 (58.6%)	50 (65%)
	Single substance	63 (26%)	45 (27.8%)	18 (23%)
	Multiple substances	31 (13%)	22 (13.6%)	9 (12%)

^1^ n (%).

**Table 2:** Clinical characteristics of study participants.

Characteristic	Category	Overall N = 239 ^1^	Favourable N = 162 ^1^	Unfavourable N = 77 ^1^
Previous drug history	New	97 (41%)	67 (41%)	30 (39%)
	Previous treatment	142 (59%)	95 (59%)	47 (61%)
Patient category	New	101 (42%)	71 (44%)	30 (39%)
	Relapse	95 (40%)	63 (39%)	32 (42%)
	Treatment failure	43 (18%)	28 (17%)	15 (19%)
Type of resistance	MONO	93 (39%)	65 (40%)	28 (36%)
	POLY	146 (61%)	97 (60%)	49 (64%)
Type of DR-TB	MDR	95 (39.7%)	68 (42.0%)	27 (35%)
	RR	127 (53.1%)	89 (54.9%)	38 (49%)
	XDR	17 (7.1%)	5 (3.1%)	12 (16%)
Comorbidity	Multiple	10 (4.2%)	8 (4.9%)	2 (2.6%)
	Single	229 (95.8%)	154 (95.1%)	75 (97.4%)

^1^ n (%). Values are presented as n (%); percentages may not total 100% (99.9%) due to rounding.

**Table 3:** Univariate Logistic Regression Analysis of Factors Associated with Treatment Outcome.

Variable	Category vs. Reference	OR (95% CI)	*p*-Value
Age group	40–49 vs. <40	0.79 (0.44–1.41)	0.474
	≥50 vs. <40	0.57 (0.29–1.12)	0.127
Gender	Male vs. Female	1.49 (0.85–2.61)	0.153
Occupation	Unemployed vs. Employed	0.64 (0.27–1.52)	0.356
Type of DR-TB	RR vs. MDR	0.93 (0.53–1.63)	0.808
	XDR vs. MDR	0.17 (0.06–0.50)	0.002
Income	Some income vs. No income	0.51 (0.27–0.95)	0.043
Comorbidity type	Single vs. Multiple	0.51 (0.24–1.10)	0.079
Type of resistance	POLY vs. Mono	0.85 (0.43–1.67)	0.578
Education	Medium vs. Low	1.02 (0.54–1.93)	0.952
	High vs. Low	1.03 (0.41–2.57)	0.958
Previous drug history	Previous treatment vs. None	0.91 (0.49–1.69)	0.724
Social history	Single substance vs. None	1.32 (0.60–2.90)	0.404
	Multiple substances vs. None	1.29 (0.49–3.39)	0.560
Patient category	Relapse vs. New	0.83 (0.44–1.56)	0.549
	Treatment failure vs. New	0.79 (0.37–1.69)	0.540

**Table 4:** Multivariable Logistic Regression Analysis of Factors Associated with Treatment Outcome (N = 239).

Variable	Category vs. Reference	Adjusted OR (95% CI)	*p*-Value
Age group	40–49 vs. <40	0.87 (0.44–1.71)	0.706
	≥50 vs. <40	0.51 (0.24–1.09)	0.089
Gender	Male vs. Female	1.55 (0.87–2.77)	0.146
Type of DR-TB	RR vs. MDR	0.91 (0.50–1.66)	0.749
	XDR vs. MDR	0.18 (0.06–0.58)	0.004
Income	Some income vs. No income	0.46 (0.23–0.92)	0.036
Comorbidity type	Single vs. Multiple	0.29 (0.06–1.38)	0.143

**Table 5:** Testing for multicollinearity.

Variable	GVIF^(1/(2Df))	Interpretation
Age group	1.01	Very low; no multicollinearity
Gender	1.02	Very low; no multicollinearity
Type of DR-TB	1.01	Very low; no multicollinearity
Income	1.04	Very low; no multicollinearity
Comorbidity type	1.05	Very low; no multicollinearity

GVIF = Generalized Variance Inflation Factor; Df = degrees of freedom. GVIF^(1/(2Df)) values < 5 indicate absence of problematic multicollinearity.

## Data Availability

Data can be requested from the corresponding authors.

## Abbreviations

The following abbreviations are used in this manuscript:

TB	Tuberculosis
DR-TB	Drug resistant tuberculosis
RR-TB	Rifampicin resistant tuberculosis
MDR-TB	Multi drug resistant tuberculosis
Pre-XDR-TB	Pre-extensively drug-resistant
XDR-TB	Extensively drug-resistant
WHO	World Health Organization
HIV	Human immunodeficiency virus
